# A Rare Form of Pott’s Disease With Multifaceted Pathological Complications

**DOI:** 10.7759/cureus.8855

**Published:** 2020-06-27

**Authors:** Ryan F Amidon, Christ Ordookhanian, Talia Vartanian, Paul Kaloostian

**Affiliations:** 1 Neuroscience, University of California, Riverside, USA; 2 Medicine, University of California, Riverside, USA; 3 Physical Medicine and Rehabilitation, University of Southern California, Los Angeles, USA; 4 Neurological Surgery, Paul Kaloostian M.D. Inc., Riverside, USA; 5 Neurological Surgery, Riverside Community Hospital, Riverside, USA

**Keywords:** central nervous system tuberculosis, cns, tuberculosis spondylitis, pott's disease, hiv, tb, tuberculosis, extraspinal, osteomyelitis, arthritis

## Abstract

While tuberculosis is globally very prevalent, especially in the developing world, tuberculosis of the central nervous system (CNS) (including Pott’s `disease) is an extremely rare occurrence for neurology/neurosurgery departments in the modern era. It is normally treated via rifampin, isoniazid, pyrazinamide, and ethambutol (RIPE) therapy with the need for surgical intervention deemed by the presence of neurological deficiency or abscess, spinal instability, or significant kyphosis. Here we describe a case of an elderly woman with Pott’s disease and a history of HIV presenting with neurologic deficiencies in both legs and an infected mass causing mid-thoracic compression and kyphotic deformity. The presence of a compromised immune system greatly complicates treatment and worsens outcomes. The patient underwent trans-thoracic corpectomy for decompression and mass removal. Spinal realignment was accomplished with an anterior graft, using the patient’s rib, preceding posterior stabilization with instrumentation. Postoperatively, the patient received RIPE therapy. Despite a compromised immune system, the full neurologic function of both legs was restored in four months.

## Introduction

With the reduced prevalence of tuberculosis (TB) in developed countries, it is easy to overlook the global impact of the disease. According to the World Health Organization (WHO), in 2018, approximately 10 million people acquired TB and it ranks among the top 10 causes of deaths globally, especially affecting the developing nations [1]. TB penetration of the central nervous system (CNS) occurs in a mere 1% of cases; however, people with HIV, or AIDS, are five times more likely to acquire CNS TB than HIV-negative people [2,3]. HIV is also prevalent in the developing world, constituting the leading cause of death in sub-Saharan Africa [4]. Pott’s disease is a form of CNS TB where the spinal cord is the site of infection. When these two conditions are coupled, positive outcomes are unlikely. Appropriate treatment of Pott’s disease with HIV is therefore very important to global health. This case highlights the successful treatment of a severe form of Pott’s disease where a uniquely located thoracic mass is responsible for severe spinal instability, spinal cord compression, and loss of motor function.

## Case presentation

**Figure 1:**
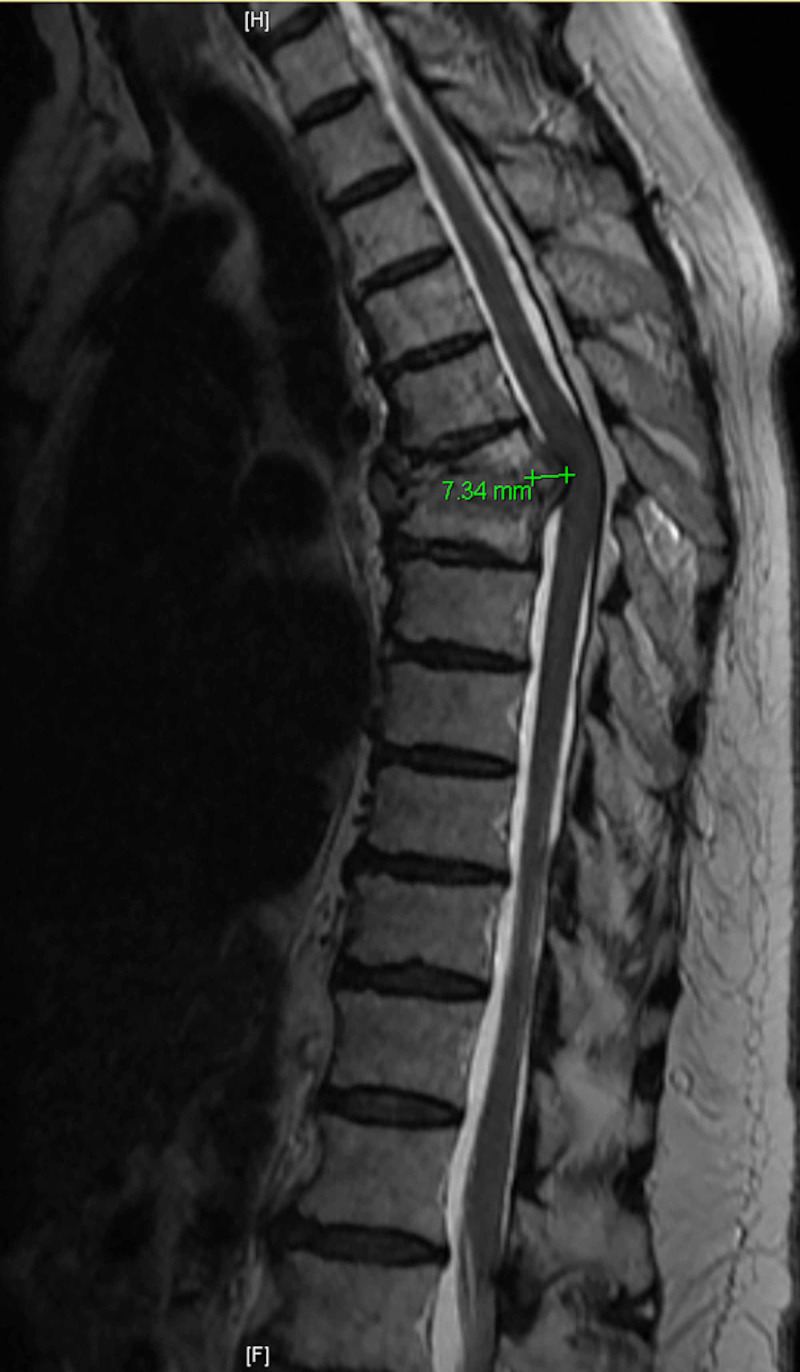
Sagittal T2 MRI of the thoracic spine indicating the presence of a severe kyphotic deformity from T6-T7 due to erosive lesion within the vertebral bodies with associated dorsal mass effect and severe spinal cord compression with signal cord change

A 60-year-old Hispanic female with a history of AIDS and treated tuberculosis of unknown initial infection date presented to the clinic following a three-month history of progressively worsening imbalance of gait, severe weakness in legs requiring wheelchair use, and leg sensory deficits. At presentation, no pulmonary *Mycobacterium tuberculosis *(MT) was visualized on chest x-ray (CXR) or computerized tomography (CT) scans. The patient presented with a severely weakened immune system as the immune cell cluster of differentiation four (CD4) count was less than 50 cells/mm^3^. Upon examination, our patient was found to have hyperreflexia in the legs with weakness and clonus. The patient retained the ability of movement but was unable to contact against gravity, which is indicative of a neurological motor exam score of 2/5 bilaterally. Upon examination, the arm function was normal and intact. Radiological studies (MRI and CT) of the thoracic spine at T6 and T7 highlighted a destructive and erosive lesion with kyphotic deformity, posteriorly extruded bony fragment/mass with severe spinal cord compression, and spinal cord signal change (Figure 1).

The patient was immediately taken into the operating room for a two-staged surgical decompression of the thoracic spinal cord.

**Figure 2:**
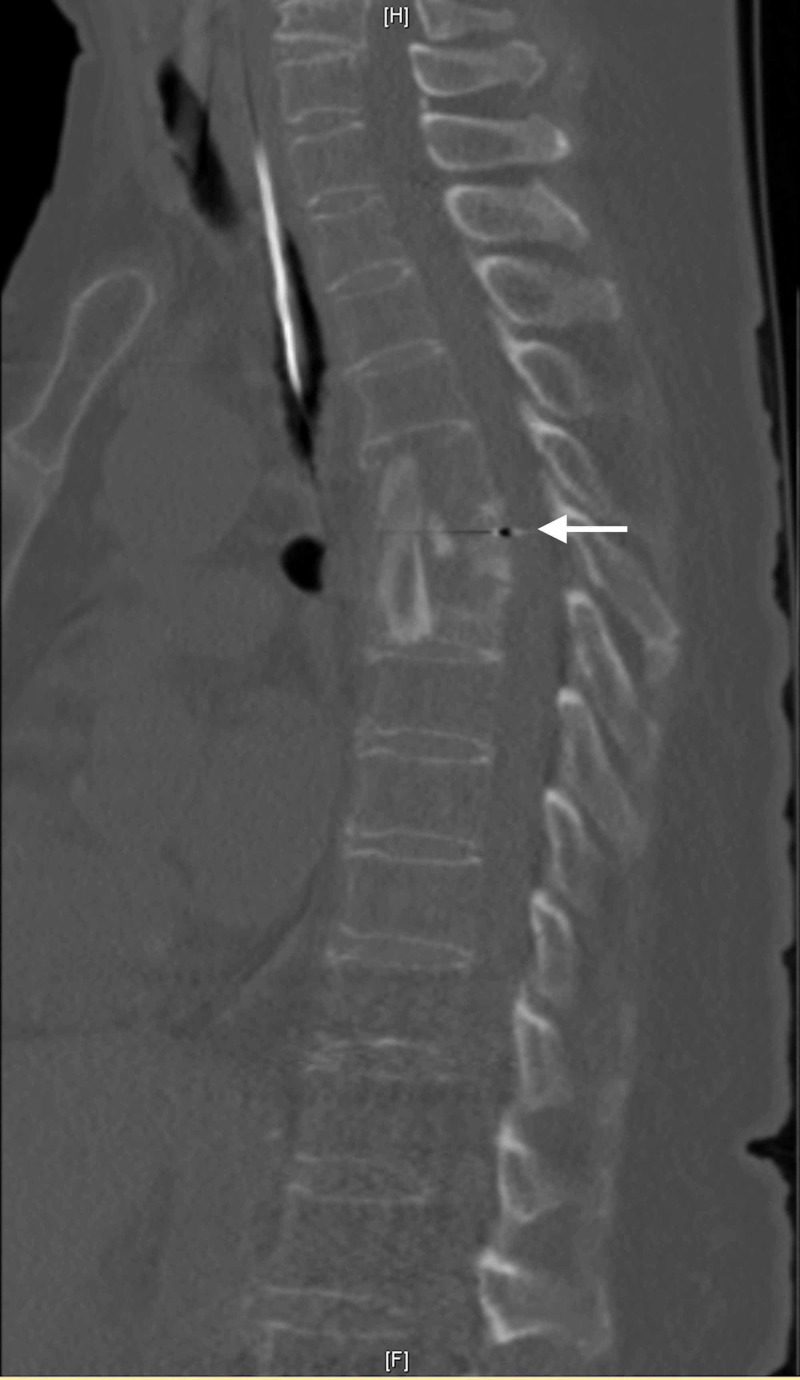
Post-operative trans-thoracic decompression and corpectomy with placement of rib autograft for resection of lesion and correction of deformity

The first stage involved trans-thoracic T5-T7 corpectomy with rib autograft placement in the corpectomy bed (Figure 2).

**Figure 3:**
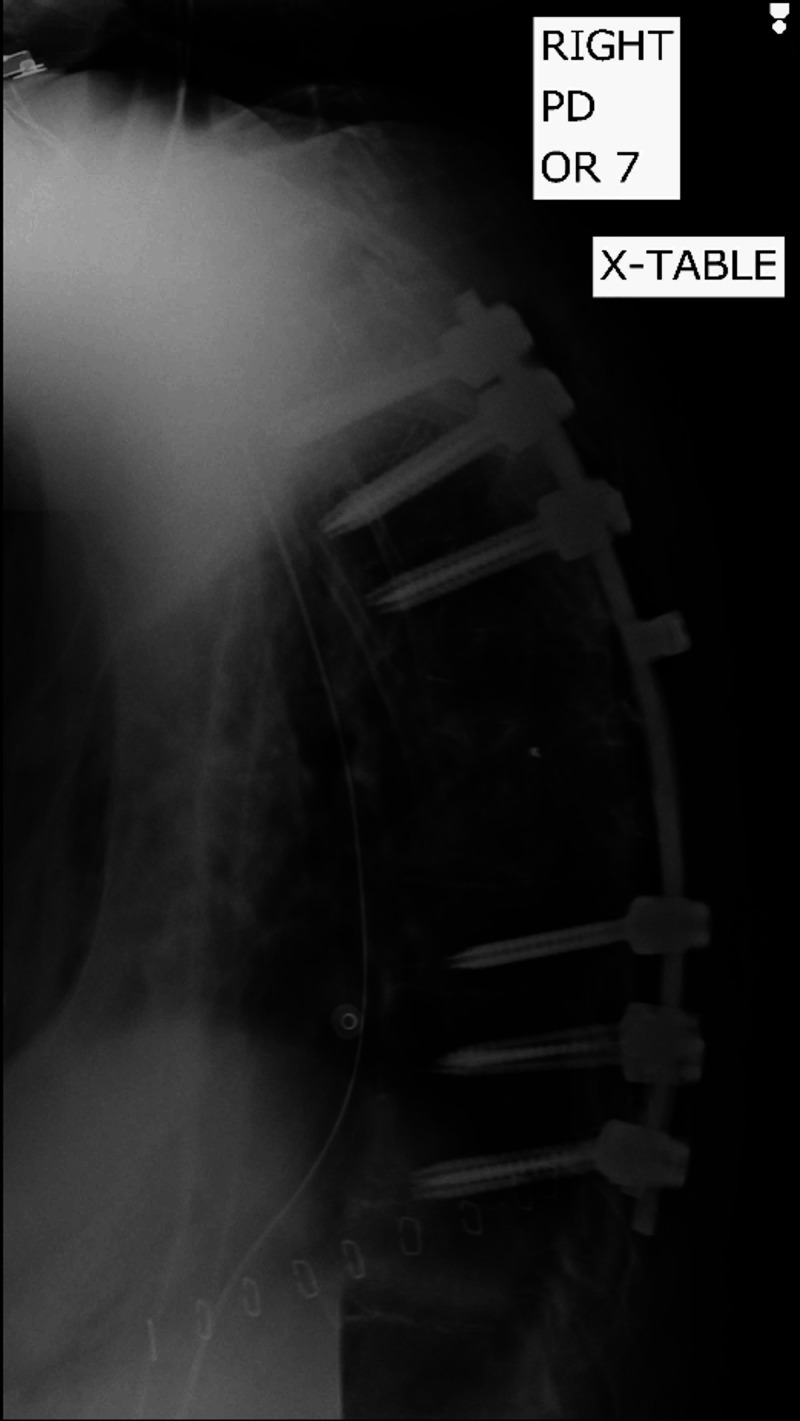
Post-operative radiological study demonstrating the instrumentation placement in the thoracic spine

Intraoperatively, the T5-T7 vertebrae were extremely soft and friable accompanied by a yellow mass within the vertebral bodies. The spinal cord was decompressed using curettes to remove the component pushing on the nearby cord. The next day, stage two of the surgical series involved posterior thoracic fusion using extensive instrumentation (Figure 3).

Post-operatively our patient recovered well and continued receiving rifampin, isoniazid, pyrazinamide, and ethambutol (RIPE) therapy which consists of: rifampin (RIF), isoniazid (INH), pyrazinamide (PZA), and ethambutol (EMB). Our patient improved strength in her legs from 2/5 pre-operatively to 4+/5 post-operatively and is now ambulatory. The patient was followed up in the clinic four months later and demonstrated appropriate strength in her legs, with a score of 5/5 and with intact sensation, signaling an unusual positive outcome for such a case.

## Discussion

TB results from MT infection, which is frequently contracted by inhaling droplets containing the bacteria (i.e., from coughing). The infection may remain dormant for decades (latent) or immediately lead to TB active symptoms. Most of the time, TB affects the lungs (pulmonary), but it may also affect other areas of the body (extrapulmonary) [1].

The primary sites of MT infection are contained when the adaptive immune system activates CD4 and CD8 T lymphocytes [5]. However, sometimes this local response fails and the infection progresses; pathogens enter the lymphatic system and travel through the bloodstream to other regions, in some cases penetrating the CNS. CNS TB is a severe form, comprising 5%-10% of extrapulmonary cases, that presents as meningitis, cerebritis, abscesses, spinal tuberculosis arachnoiditis, or tuberculomas. Risk factors for MT entering the CNS include malnutrition, alcoholism, malignancies, age, and immunosuppressed states [2].

When TB involves the bones and/or joints, it is referred to as skeletal TB, comprising 10%-35% of extrapulmonary cases. It should be noted that any bone is susceptible to being infected by MT. Skeletal TB cases can be categorized into the arthritis form, the osteomyelitis form, or the spondylitis form (also known as Pott’s disease and is the most common of the three). In Pott’s disease, the bones typically involved are the lower thoracic and upper lumbar, and inflammation of intervertebral joints signals its progression. Beginning at one vertebral level, adjacent vertebrae may eventually become involved when the intervertebral disc space is infected. Such noncontiguous spinal disease (involvement at more than one level) is relatively rare but remains a possibility. Vertebral narrowing and collapse can occur, potentially resulting in cord compression and subsequent paraplegia, particularly when the mid-thoracic is compressed [5].

When diagnosing Pott’s disease, several differential diagnoses exist: degenerative disc and facet joint disease, spondyloarthropathy, pyogenic spinal infection, vertebral body collapse from osteopenia, and malignancy - their clinical features are similar, so radiography (CT and MRI) is invaluable for accurate diagnosis [5]. Cerebrospinal fluid (CSF) examination may also be used: Look for decreased glucose and increased protein concentrations. An increase in adenosine deaminase (ADA) concentration may also be used; however, this could also be indicative of another infection and therefore should not be relied upon [6].

When treating TB, early diagnosis is crucial. Standard treatment is a mix of antibiotics, generally including rifampin, isoniazid, pyrazinamide, and ethambutol (together known as RIPE therapy). The key to improved prognosis is the sterilizing activity of these drugs to eliminate the MT remaining after the first days of this therapy [7]. As antibiotics have been used to treat TB for many years, it is natural that antibiotic-resistant TB strains have become major threats: over 3% of new cases and nearly 20% of old cases were found to be antibiotic-resistant in 2018 [1]. Treating antibiotic-resistant TB requires the use of drugs that the strain has not yet encountered. Fortunately, many new drugs are emerging or are in development that may confront drug-resistant TB. Surgery is appropriate when the patient has neurological deficits (i.e., paraplegia), progressively diminishing neurological functions while receiving treatment, spinal kyphosis greater than 40 degrees, or a chest wall cold abscess [5]. In our case, neurological deficits and kyphosis were both indications.

Of the numerous risk factors for TB, HIV infection is particularly significant [8]. Approximately 251,000 TB-related deaths occurred among those infected with HIV [1]. Of TB cases with HIV coinfection, 40% are extrapulmonary [3].

HIV is a retrovirus and antiretroviral drugs are administered for treatment (antiretroviral treatment, or ART). This treatment can be complicated by the onset of immune reconstitution inflammatory syndrome (IRIS), where the immune system displays an excessive inflammatory response to an infection, enhancing its severity. IRIS may reactivate latent TB or progress active TB [6]. Patients with HIV who have already undergone ART will receive RIPE therapy for TB treatment. However, for patients with HIV that have not yet undergone ART, there is controversy regarding when it should be initiated.

In 2011, Dr. Christina Nelson and Dr. Joseph Zunt recommended an early start to ART for patients with HIV and TB, especially those with CD4 cell counts less than 50 cells/mm^3^, which indicates a severely weakened immune system as the normal range is above 500 cells/mm^3^ [3]. At this time, the WHO recommended ART after beginning RIPE therapy, regardless of CD4 cell count, but they did not provide a recommended period between the initiation of RIPE therapy and the beginning of ART. Nelson and Zunt brought up several studies in defense of early ART initiation: The first demonstrates similar outcomes for patients given immediate and delayed (two months) ART. The second demonstrates decreased mortality for patients given ART within four weeks of RIPE therapy. However, both studies observed increased rates of IRIS in patients with CD4 cell counts less than 50 cells/mm^3 ^[9]. The third study demonstrates increased survival for patients receiving ART within two weeks of RIPE therapy compared to those receiving it within eight weeks. A limitation of these studies is that the patients did not have CNS TB [3]. The American Thoracic Society, the Centers for Disease Control and Prevention, and the Infectious Diseases Society of America published guidelines in 2016 recommending that ART begin within 8-12 weeks or two weeks for patients with CD4 cell counts under 50 cells/mm^3^ [10]. Taking into account the aforementioned guidelines, in 2019, Dr. John Leonard recommended generally delaying ART by eight weeks after beginning RIPE therapy regardless of the CD4 cell count. This was supported by a study providing evidence that early initiation of ART (within two weeks) following RIPE therapy is associated with increased mortality and worsened outcomes [6]. A consensus surrounding this timing is currently absent and recommendations will continue to evolve as more research emerges.

The difficulties that stem from diagnosing and treating CNS TB with HIV coinfection stem from having to consider a variety of differential diagnoses, drug-drug interactions, the timing of ART, the potential for IRIS, abnormal CSF characteristics, and worsened outcomes [8]. Furthermore, patients with low CD4 cell counts (less than 50 cells/mm^3^) are less likely to have abnormal CSF results upon examination, complicating the diagnosis of CNS TB [3]. Other factors contributing to the high mortality rate in patients infected with both HIV and CNS TB include increased severity of symptoms upon presentation, low CD4 cell count, and a higher likelihood of harboring multi-drug resistant TB [3]. For instance, rifampin-resistant and isoniazid-resistant strains are associated with HIV coinfection, complicating TB treatment. Under these conditions, the drugs administered and their associated dosages are modified.

Our patient’s advanced age and history of both TB and HIV represented significant risk factors for CNS TB. Her history, presentation of neurologic deficiency (imbalance of gait, paraplegia, hyperreflexia with weakness and clonus in the legs, and sensory deficits), and radiographic evidence led to the diagnosis of Pott’s disease. Significant kyphosis, cord compression, and mid-thoracic lesion were proper indicators for surgical intervention. The paraplegia developed from mid-thoracic compression. Decompression and stabilization were, therefore, appropriate approaches to restoring neurologic function. Decompression was achieved through a trans-thoracic T5-T7 corpectomy with rib autograft placement in the corpectomy bed. It is economical and just as efficient to use an autograft from the patient’s rib, which was intraoperatively removed for exposure, to restore alignment as using foreign body titanium cages that are expensive and at risk for infection. A yellow mass on gross pathology was observed within the vertebral bodies removed. Thoracic fusion with instrumentation was then conducted for stabilization.

Post-operative treatment included the initiation of RIPE therapy. The patient had already undergone ART, so timing of these treatments was not an obstacle. The patient was referred to primary care for low CD4 cell count treatment and for starting prophylactic antibiotics (azithromycin and clarithromycin) to prevent *Mycobacterium avium* complex (MAC) disease when the CD4 count decreases below 50 cells/mm^3^. While it is apparent that HIV patients with a history of TB are less likely to acquire MAC disease than those without prior TB, it can still occur. It is hypothesized that previous infection with TB may enhance immunity to mycobacteria, reducing the chance of *Mycobacterium avium* infection [11].

As a result of our intervention, our patient fully regained the neurologic function of her legs after four months. Our success provides useful lessons for treating patients with Pott’s disease and HIV in the future. Suspicion of CNS TB must be maintained while diagnosing a patient at risk for such a development as early detection and intervention are critical for recovery. Pott’s disease in combination with HIV infection presents an exceedingly rare case within medicine, as well as a complex one, which frequently results in poor outcomes. This case is especially significant to global medicine as TB and HIV are both very prevalent and deadly in the developing world.

## Conclusions

Due to the rare nature of Pott’s disease in combination with HIV infection, there is still much to be understood regarding optimal treatment. This case illustrates that when a spinal mass causing symptomatic cord compression and instability is discovered, it is imperative to decompress the compressive pathology first to remove pressure off the spinal cord. Subsequently, a posterior approach may then be undertaken with the goal of stabilization of the anterior construct. Using an autograft taken from the patient’s rib (intraoperatively resected for exposure) for the creation of thoracic alignment is advantageous when compared to titanium grafts and cages, which are not only expensive but increase risk of infection. Since HIV is present, such an infection could prove deadly. Our intervention resulted in an unusually positive post-operative recovery despite overwhelming immune system compromise. We are hopeful that our case will provide the needed insight into the treatment of complex spinal pathologies along with valuable learning lessons.
